# Effective Use of Clozapine in Managing Treatment-Resistant Conduct Disorder in an Adolescent Patient: An Unconventional Approach

**DOI:** 10.1192/j.eurpsy.2024.931

**Published:** 2024-08-27

**Authors:** E. Yerlikaya Oral

**Affiliations:** Department of Child and Adolescent Psychiatry, University of Health Sciences, Bakirkoy Prof. Dr. Mazhar Osman Research and Training Hospital for Psychiatry, Neurology and Neurosurgery, Istanbul, Türkiye

## Abstract

**Introduction:**

This case report elaborates an unconventional approach to the management the use of clozapine, typically used in treatment-resistant Schizophrenia, in a 17-year-old female patient with treatment-resistant Conduct Disorder, Attention Deficit and Hyperactivity Disorder and Intellectual Disability.

**Objectives:**

The aim is to demonstrate the potential effectiveness and applicability of clozapine in treating severe, treatment-resistant behavioral problems associated with Conduct Disorder, even without the presence of psychosis, by detailing the clinical course, treatment strategy and outcome of this unique case.

**Methods:**

A 17-year old female patient was referred to inpatient clinic due to escalating aggression towards her family members and risky sexual behaviors despite undergoing treatments before as risperidone, haloperidole, olanzapine, lithium, clonidin or aripipirazole. She was running away from home repeatedly and under the risk of sexual abuse. After comprehensive clinical and psychopathological assessment, a decision was made to initiate treatment with clozapine, closely monitoring the patient for adverse effects, and assessing its impact on the patient’s aggressiveness and other behavioral problems. During her stay, clozapine dose titrated to 250 mg/day in addition to her current treatment as amisulpiride 800 mg/day and valproic acid 500 mg/day. Atropine solution as mouthwash used for salivary hipersecretion. No other side effects observed. Cognitive and behavioural therapy interventions made for anger management and impulsivity. Also focused on family-based interventions about establishing healthy boundaries.

**Results:**

A significant reduction in aggressive behavior was noted under the treatment of clozapine. The patient’s overall conduct and interaction with family improved remarkably. The treatment was well-tolerated except sialorrhea, leading to a successful integration back into her family and community.

**Conclusions:**

This case highlights the potential of clozapine as a viable treatment option for managing severe, treatment-resistant behavioral problems in patients with conduct disorder, even in the absence of psychosis. A randomized-controlled trial showed that clozapine was more effective than risperidone in conduct externalization factors, delinquency trait and global functioning in children and adolescents(Juárez-Treviño *et al. Clin Psychopharmacol Neurosci*. 2019;17(1):43-53). While it necessitates careful monitoring due to its side-effect profile, this unconventional use of clozapine may open up new avenues for the management of treatment-resistant conduct disorder, thereby improving patient outcomes and quality of life. Further controlled studies are warranted to ascertain the safety and efficacy of this approach.

**Disclosure of Interest:**

None Declared

